# Looking forward to the new year

**DOI:** 10.1016/j.patter.2023.100916

**Published:** 2024-01-12

**Authors:** Andrew L. Hufton

**Affiliations:** Editor-in-Chief, *Patterns*

2023 was a year where we marveled at the power of artificial intelligence (AI) to chat, write, and create art and where many fields continued to be transformed by rapidly advancing deep learning and large language model methods. At the same time, it was a year where we, both scientists and society at large, grappled like never before with the ethical implications of these technologies: their ability to drive forward research and to advance a variety of technology applications but also their potential for abuse and economic disruption and to create new sources of inequality.

At *Patterns*, we published work showing that GPT detectors were biased against non-English speakers (Liang et al.), as well as work exploring ethical frameworks for AI (McCradden et al.; Corrêa et al.) and sharing different viewpoints on the potential moral standing of future AIs (Marshall; Schwitzgebel). These and other papers looking at the ethics and social impact of data science are presented in our ongoing special collection on responsible and accountable data science.

Going into 2024, we look forward to publishing more top research works in the fields of machine learning and artificial intelligence research. In these fields, we will be selecting in particular for papers that apply such methods creatively to solving new problems, that reveal and validate new insights into our natural world, or that explore in detail how such tools can interact with human systems to meaningfully improve decisions or outcomes. At the same time, we will be seeking to diversify our content to ensure that we are covering data science research in the broadest sense possible, by expanding our coverage of topics in data management and curation, by inviting compelling work from fields across all domains of science, and by looking critically at societally relevant topics, such as the energy consumption of advanced computation. To support this diversification of our content, we will be announcing in the first half of 2024 a major expansion and renewal of our advisory board.

As another part of this effort to broaden our content, we are delighted to be relaunching our creations format—short essays highlighting intersections between art and data science. To learn more, and to submit a proposal, please see our call for papers page and the announcement entitled “Transforming Data into Art”.

To better help support our broad and diverse set of authors, we are also working on improving the quality of our journal information pages. As one small step in this process, we recently updated our guidance for authors preparing a research article and shared a new template for LaTeX users that will aid manuscript preparation. We have also recently updated our collection index page to help readers browse papers from our different content types. More improvements and updates will be made in the coming months.

As part of this process, we will be phasing out the use of data science maturity levels (DSML). DSML ratings were designed to provide transparency about how ready a data science method was for real-world-use, and to help readers explore papers at different stages of development, from ideation to real-world testing. In practice, however, this five-step scale has proven difficult to apply consistently, especially to papers that do not focus specifically on methods development.

These changes are part of our commitment to progressively improving the journal. We welcome your feedback during this journey. And, we look forward to seeing what the new year brings and to sharing with you the latest in data science and data-heavy research.

